# Frequency and Outcomes of Acute‐on‐Chronic Liver Failure in Nonelective Cirrhotic Patients Admitted to a Brazilian ICU: A Single‐Center Retrospective Study

**DOI:** 10.1155/cjgh/9728104

**Published:** 2025-08-26

**Authors:** Maria Eduarda Chaves Soares, Liana Codes, Bianca Sampaio de Carvalho, Amanda Caroline Silveira e Silva, Myriam Sofia Angeli Guimarães de Oliveira, Fabiola Santos Sousa, Mariana Rebouças de Calasans, Jade de Oliveira Santana, Lucas Celes Dominguez, Paulo Lisboa Bittencourt

**Affiliations:** ^1^ Unit of Gastroenterology and Hepatology, Portuguese Hospital, Salvador, Bahia, Brazil; ^2^ Department of Gastroenterology, Bahiana School of Medicine and Public Health, Salvador, Bahia, Brazil, bahiana.edu.br

**Keywords:** acute-on-chronic liver failure, cirrhosis, critical care, organ dysfunction, outcome, prognosis

## Abstract

**Introduction:** Acute‐on‐chronic liver failure (ACLF) is a severe complication of cirrhosis characterized by acute decompensation (AD), organ failure(s), and high mortality.

**Aims:** To investigate the frequency and the clinical course of ACLF in intensive care unit (ICU) patients at different time points, using CLIF‐C and NACSELD criteria as well as to assess their influence on mortality.

**Methods:** Patients admitted with AD with and without ACLF were retrospectively evaluated.

**Results:** 595 patients (443 males, mean age: 66.6 ± 12.0 years) were admitted due to AD (*n* = 381) or ACLF (*n* = 214). According to the CLIF‐C criteria, 119 patients (20%) had ACLF Grade I, 63 (10.6%) had ACLF Grade II, and 32 (5.4%) had ACLF Grade III at admission. Using the NACSELD, 155 patients (26.1%) had ACLF at admission. Infection was the main factor associated with ACLF at admission (*n* = 57; 27%, *p* = 0.001). In total, 104 (17.5%) patients died during hospitalization. ACLF grade at admission (OR: 4.6; 95% CI: 2.45–8.67; NS: 0.0001), use of vasopressors (OR: 3.83; 95% CI: 1.15–12.7; NS: 0.02), and CLIF‐C ACLF (OR: 1.12; 95% CI: 1.06–1.19; NS: 0.0001) were independently associated with in‐hospital mortality. The improvement in organ dysfunction after 7 days of intensive care was associated with a reduction in the risk of in‐hospital mortality compared to the 3‐day period (OR: 0.098; 95% CI: 0.047–0.204 vs. 0.253; 95% CI: 0.127–0.504; *p* < 0.00001, respectively).

**Conclusion:** ACLF is associated with significant mortality in ICU patients, the CLIF‐C criteria appear to be more effective for prognostic assessment than NACSELD, and 7 days of intensive care may improve clinical outcomes.

## 1. Introduction

Acute‐on‐chronic liver failure (ACLF) has been characterized by the onset of organ failure (OF) in patients with cirrhosis [1–4]. There are currently several definitions for ACLF in the literature [4]. The two most employed criteria for ACLF in daily practice were those proposed by the Chronic Liver Failure Consortium (CLIF‐C), endorsed by the European Association for the Study of the Liver (EASL) and the North American Consortium for the Study of End‐Stage Liver Disease (NACSELD) [2, 3]. CLIF‐C criteria were prospectively developed to recognize hospitalized patients with cirrhosis and OF leading to more than 15% 28‐day mortality [2], whereas the NACSELD criteria were proposed to characterize cirrhotic patients with more advanced OF usually in the intensive care unit (ICU) for organ support with a much worse prognosis [3].

The course of ACLF was shown to fluctuate during hospitalization. In this respect, subjects who had reversal of ACLF with ICU support were shown to have better outcomes when compared to their counterparts with persistent ACLF [5].

Up two now, few reports have investigated the frequency and outcomes of those cirrhotic patients with ACLF in Brazil [6–9]. Overall, 24%–30% of those patients had ACLF when assessed with CLIF‐C criteria in the first 24 h of admission [6, 8, 9]. Prognosis was shown to be poor, particularly in those patients with either persistent or with advanced grades of ACLF [7–9]. In those patients, employment of liver specific scores in the ICU based on the presence of ACLF such as CLIF‐C OF, CLIF–sequential OF assessment (SOFA), and CLIF‐C ACLF was shown to have a better accuracy to predict mortality when compared to general ICU scoring systems such as APACHE II and SOFA [10, 11].

Few data are available regarding the frequency and outcomes of cirrhotic patients admitted to the ICU with ACLF defined either with CLIF‐C or NACSELD criteria.

The aims of the present study were to investigate the frequency of ACLF at different time points in patients with cirrhosis admitted to the ICU using CLIF‐C and NACSELD criteria, as well as to assess the influence of the clinical course of ACLF and other clinical and laboratory variables on ICU mortality and 28‐day survival.

## 2. Patients and Methods

All patients with cirrhosis admitted on a nonelective basis to the gastroenterology and hepatology unit of the Portuguese Hospital of Salvador, Bahia, Brazil, between January 2012 and June 2024, were retrospectively evaluated. This hospital is a referral center for liver transplantation as well as for the management of critically ill patients with cirrhosis. The diagnosis of cirrhosis was based on clinical, biochemical, and imaging findings, as well as liver histology, whenever liver biopsy results were available. Unless otherwise specified, the term ACLF refers to ACLF as defined by the CLIF‐C criteria.

The inclusion criteria were hospital admission due to acute decompensation (AD) of cirrhosis, which could be caused by one or more of the following: ascites, gastrointestinal bleeding, hepatic encephalopathy, and infection. All cirrhotic patients admitted for elective procedures such as intra‐arterial chemoembolization for hepatocellular carcinoma, or those with severe comorbidities (HCC beyond Milan’s criteria, extrahepatic malignancy, infection by the human immunodeficiency virus, chronic renal failure requiring dialysis, and heart failure classified as Class III or Class IV by the New York Heart Association), or previous solid organ transplant were excluded from the study.

Data regarding demographics; presence of comorbidities; etiology of cirrhosis and clinical features responsible for ICU admission; and baseline laboratory parameters including leukocyte counts, platelets, international normalized ratio (INR), total bilirubin, serum sodium, albumin, and creatinine levels were collected. Prognostic scores were calculated within 24 h of ICU admission. Occurrence of ACLF either by CLIF‐C or NACSELD criteria was assessed, as previously described [2, 3], at baseline in the first 24 h of ICU admission as well as three (D3) and seven (D7) days thereafter. The clinical course of patients with ACLF was reckoned. ACLF resolution was characterized by a return to ACLF 0. Improvement was indicated by a reduction in the ACLF score from 3 to 2, 3 to 1, or 2 to 1. Worsening was characterized by a change in ACLF grade from 1 to 2 or 3, or from 2 to 3. A steady course was indicated by the absence of any change in ACLF grade. The risk of in‐hospital death was evaluated according to different time points of hospitalization (first 24 h, 3 days, or 7 days of intensive support). Patients were followed up until death or up to 28 days after discharge. The primary outcome was in‐hospital mortality or transplant‐free survival.

The study adhered to the Declaration of Helsinki and was approved by the Ethics Committee for Research at Hospital Português in Salvador, Bahia.

### 2.1. Statistical Analysis

Dichotomous variables are presented in text and tables as numbers and percentages and continuous variables were expressed as mean ± standard deviation (SD) or as median and interquartile range, respectively, whether the distribution was normal or skewed. Demographic, clinical, and laboratory variables were compared using the chi‐square test or Fisher’s test for categorical variables or Student’s *t*‐test or the Mann–Whitney *U* test for continuous variables when appropriate. A logistic regression model was fitted to select the best subset of predictors of hospital mortality. Factors that showed a significance level of 0.10 in the univariate analysis were selected for the multivariate analysis. The final model was constructed using a stepwise forward method, based on improvements in model likelihood ratios. A *p* value < 0.05 was considered significant. Statistical analyses were performed with the Statistical Package for Social Sciences (SPSS Inc., Chicago, IL, USA), Version 21.0 for Windows.

## 3. Results

A total of 595 consecutive patients (443 males, mean age: 66.6 ± 12.0 years) were admitted to the ICU due to AD (*n* = 381) of cirrhosis or ACLF (*n* = 214) according to the CLIF‐C criteria. Table 1 shows demographics, clinical features, and outcomes of these patients. AD occurred due to ascites (*n* = 209; 35%), encephalopathy (*n* = 164; 27.6%), infection (*n* = 113; 19%), and variceal hemorrhage (*n* = 109; 18.3%). Alcohol‐related liver disease was the main etiology (*n* = 192; 32%). According to the CLIF‐C criteria, 119 patients (20%) had ACLF Grade I, 63 (10.6%) had ACLF Grade II, and 32 (5.4%) had ACLF Grade III at admission. Using the NACSELD definition, 155 patients (26.1%) had ACLF at admission. Mechanical ventilation, dialysis, and vasopressors were required upon ICU admission in 23 (3.9%), 33 (5.5%), and 43 (7.2%) of the cases, respectively. The ICU length of stay was 4 (2–7) days.

**Table 1:** Demographics, clinical features, and outcomes of cirrhotic patients at the time of admission to the ICU.

Characteristics	All patients (*n* = 595)	CLIF‐C criteria	
		Cirrhosis with ACLF (*n* = 214)	Cirrhosis with AD (*n* = 381)
Age (years)	66.6 ± 12.1	64.5 ± 12.5	67.8 ± 11.6
Male sex	443 (74.4)	156 (72.9)	287 (75.3)
Decompensation			
Ascites	209 (35.1)	79 (36.9)	130 (34.1)
Hepatic encephalopathy	164 (27.6)	48 (22.4)	116 (30.4)
Variceal bleeding	109 (18.3)	30 (14.0)	79 (20.7)
Bacterial infection	113 (19.0)	57 (26.6)	56 (14.7)
MELD score	16.1 ± 7.8	22.2 ± 7.9	12.5 ± 4.9
ACLF by NACSELD criteria		104 (48.6)	
ACLF by CLIF‐C criteria		214 (100)	
Grade I		119 (55.6)	
Grade II		63 (29.4)	
Grade III		32 (15.0)	
CLIF‐C ACLF		90.0 ± 8.4	
CLIF‐C OF		8.9 ± 1.5	
Laboratory data			
INR^1^	1.6 (1.4–2.0)/0.9–6.1	1.7 (1.3–2.5)/1.0–6.1	1.6 (1.4–1.8)/0.9–2.8
Bilirubin (mg/dL)^1^	1.7 (0.9–3.1)/0.1–36.7	1.7 (0.7–4.9)/0.1–36.7	1.8 (1.0–2.7)/0.1–11.6
Creatinine (mg/dL)^1^	1.0 (0.7–1.7)/0.2–9.6	2.1 (1.0–3.2)/0.3–9.6	0.9 (0.7–1.1)/0.2–6.2
Leucocytes (x10^9^/L)^1^	5400 (3773–8378)/1340–41740	6140 (3900–9500)/1530–36000	5200 (3720–7900)/1340–41740
ICU length of stay^1^	4.0 (2.0–7.0)/1.0–40.0	6.0 (3.0–9.0)/1.0–25.0	4.0 (2.0–6.0)/1.0–40.0
ICU mortality	104 (17.5)	74 (34.6)	30 (7.9)
28‐day postdischarge survival	441 (74.1)	121 (56.5)	320 (84.0)

*Note:* CLIF‐C, Chronic Liver Failure Consortium.

Abbreviations: ACLF, acute‐on‐chronic liver failure; ICU, intensive care unit; INR, international normalized ratio; MELD, Model for End‐Stage Liver Disease; NACSELD, North American Consortium for the Study of End‐Stage Liver Disease; OF, organ failure.

^1^Expressed by median (25th–75th)/min–max.

Table 2 summarizes the clinical and laboratory characteristics of patients with and without ACLF at the time of ICU admission. Infection was identified as the main risk factor for ACLF. Patients with ACLF exhibited significantly higher white blood cell counts; lower mean arterial pressure; and elevated levels of bilirubin, creatinine, and INR compared to those without ACLF (*p* < 0.0001). In this cohort, 104 (17.5%) patients died during hospitalization. In‐hospital mortality was significantly associated (*p* < 0.001) with the severity of ACLF at admission.

**Table 2:** Clinical features and laboratory data according to ACLF grade by CLIF‐C criteria at the time of admission to the ICU.

Characteristics	ACLF grade at time of admission				*p* value
	No ACLF (*n* = 381)	ACLF‐1 (*n* = 119)	ACLF‐2 (*n* = 63)	ACLF‐3 (*n* = 32)	
Decompensation					< 0.001
Ascites	130 (34.1)	48 (40.3)	27 (42.9)	4 (12.5)	
Encephalopathy	116 (30.4)	24 (20.2)	18 (28.6)	6 (18.8)	
Variceal bleeding	79 (20.7)	18 (15.1)	5 (7.9)	7 (21.9)	
Bacterial infection	56 (14.7)	29 (24.4)	13 (20.6)	15 (46.9)	
MELD score	12.5 ± 4.9	21.2 ± 6.7	21.7 ± 8.4	26.5 ± 10.9	< 0.001
Laboratory data					
INR^1^	1.6 (1.4–1.8)/0.9–2.8	1.7 (1.4–2.5)/1–5.6	1.5 (1.3–2.7)/1.0–6.1	2.0 (1.4–2.9)/1.1–5.9	< 0.001
Bilirubin (mg/dL)^1^	1.8 (1.0–2.7)/0.1–11.6	1.7 (0.8–3.8)/0.2–22.6	1.2 (0.3–10.3)/0.1–36.7	3.2 (0.9–7.9)/0.2–32.3	< 0.001
Creatinine (mg/dL)^1^	0.9 (0.7–1.1)/0.2–6.2	2.1 (1.0–2.8)/0.4–9.6	2.3 (1.1–3.8)/0.4–8.7	2.6 (1.2–3.5)/0.3–6.3	< 0.001
Leucocytes (× 10^9^/L)^1^	5200 (3720–7900)/1340–41740	5750 (3600–8700)/2100–33900	5550 (3810–9935)/2100–36000	9600 (6978–15885)/2540–31080	0.023
ICU mortality	30 (7.9)	29 (24.4)	22 (34.9)	23 (71.9)	< 0.001

*Note:* CLIF‐C, Chronic Liver Failure Consortium.

Abbreviations: ACLF, acute‐on‐chronic liver failure; ICU, intensive care unit; INR, international normalized ratio; MELD, Model for End‐Stage Liver Disease; NACSELD, North American Consortium for the Study of End‐Stage Liver Disease; OF, organ failure.

^1^Expressed by median (25th–75th)/min–max.

**Figure 1:**
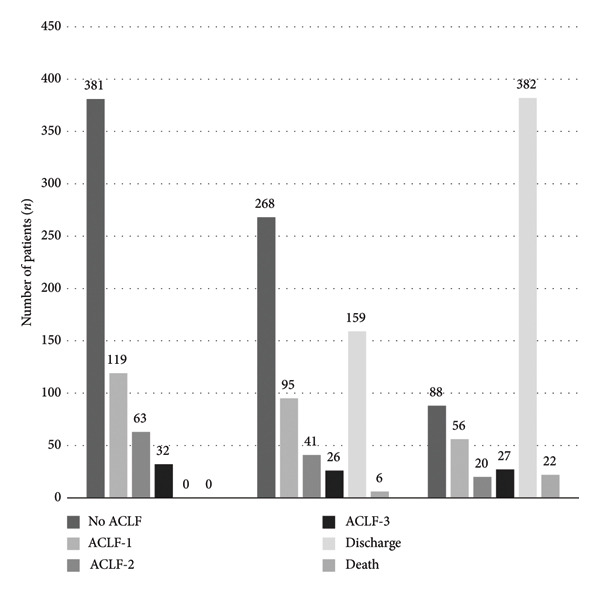
Number of patients, by ACLF grade and outcomes, at admission (D0), after 72 hours (D3), and after 7 days (D7) in the intensive care unit.

Figure 1 shows data of patients admitted to the ICU with or without ACLF and their outcomes in the first 24 h of admission (D0) and after 3 or 7 days of intensive support.

Patients with ACLF exhibited a variable clinical course. Between admission (Day 0) and Day 3, 40% of patients showed improvement in their ACLF grade, while 47% experienced stabilization and 13% worsening, whereas between admission (Day 0) and Day 7, 44% of patients showed improvement in their ACLF grade, while 36% had stabilization and 20% experienced worsening.

Among the patients admitted with ACLF‐1, 44% showed stability or improvement between Day 0 and Day 3. In patients with initial ACLF‐ 2 and 3, 35% and 15% showed improvement or stability during this same period (Day 0–3).

Higher frequency of either stabilization or improvement was observed after 7 days of ICU support, 66%, 62%, and 18%, respectively, for those subjects admitted with ACLF‐ 1, 2, and 3.

Patients who improved their ACLF grade during either 3 (16% vs. 45%, *p* < 0.0001) or 7 days (11% vs. 56%, *p* < 0.0001) of ICU stay had a significantly lower in‐hospital mortality rate compared to their counterparts with stabilization or worsening of ACLF. During the first 7 days of hospitalization, 9.2% of patients admitted with only AD developed ACLF.

On univariate analysis (Table 3), infection, occurrence of ACLF according to either CLIF or NACSELD criteria, ACLF grade, worsening of ACLF in D3 and D7, MELD score, bilirubin and creatinine levels and INR at admission, use of norepinephrine, and mechanical ventilation upon ICU admission, as well as the CLIF‐OF and CLIF‐C ACLF scores, were significantly associated with ICU mortality. However, only ACLF grade at admission (OR: 4.6; 95% CI: 2.45–8.67; *p* < 0.0001), use of vasopressors (OR: 3.83; 95% CI: 1.15–12.7; *p* = 0.02), and CLIF‐C ACLF at ICU admission (OR: 1.12; 95% CI: 1.06–1.19; *p* < 0.0001) were independently associated with death in the ICU.

**Table 3:** Univariate and multivariate variables associated with ICU mortality in subjects with ACLF.

	Univariate analysis			Multivariate analysis		
	OR	95% CI	*p* value	OR	95% CI	*p* value
Infection as decompensation	4.48	2.49–8.1	< 0.001			
MELD score at admission	1.14	1.09–1.16	< 0.002			
ACLF by NACSELD criteria at admission	3.4	2.19–5.29	< 0.001			
ACLF by CLIF‐C criteria at admission	12.69	1.68–95.64	< 0.001			
ACLF grade by CLIF‐C criteria at admission	28.82	12.2–68.04	< 0.001	4.6	2.45–8.67	< 0.001
CLIF‐C ACLF at admission	1.16	1.12–1.20	< 0.001	1.12	1.06–1.19	< 0.001
CLIF‐C OF at admission	1.77	1.53–2.05	< 0.001			
Use of vasopressors at admission	8.32	4.34–15.9	< 0.001	3.83	1.15–12.7	0.020
Mechanical ventilation at admission	5.69	2.44–13.3	< 0.001			
Serum bilirubin at admission	1.13	1.08–1.18	< 0.001			
Serum creatinine at admission	1.21	1.06–1.39	0.006			
INR at admission	2.68	1.96–3.69	< 0.001			
Clinical decline from D0 to D3	3.07	0.92–10.3	0.070			
Clinical decline from D3 to D7	9.85	5.21–18.6	< 0.001			

*Note:* CLIF‐C, Chronic Liver Failure Consortium.

Abbreviations: ACLF, acute‐on‐chronic liver failure; ICU, intensive care unit; INR, international normalized ratio; MELD, Model for End‐Stage Liver Disease; NACSELD, North American Consortium for the Study of End‐Stage Liver Disease; OF, organ failure.

The improvement in organ dysfunction after 7 days of intensive care support was associated with a greater reduction in the risk of in‐hospital mortality compared to the 3‐day period (OR: 0.098; 95% CI: 0.047–0.204 vs. 0.253; 95% CI: 0.127–0.504; *p* < 0.00001, respectively). Overall, 441 patients (74.1%) achieved 28‐day transplant‐free survival.

## 4. Discussion

This study demonstrated a high prevalence and severity of ACLF among cirrhotic patients admitted to the ICU. ACLF improvement in some patients underscores the importance of prompt interventions and appropriate intensive support. Finally, the CLIF‐C criteria appear to be more effective in the prognostic assessment of these patients.

Several scores have been developed in recent years to predict the outcomes of patients with ACLF [11]. As the genetic background, causes of cirrhosis, and types of precipitating events vary considerably worldwide, we can speculate that the performance of each score may depend on the studied population. In the current study, we found better performance for the CLIF‐C compared to NACSELD. Li F and Thuluvath also showed that the CLIF‐C criteria have a better prognostic accuracy [12].

Some studies have suggested that ACLF is more prevalent among males, who also have higher mortality rates than females. Age has also appeared to be a predictor of survival in severe forms of the disease [13, 14]. In our study, gender and age did not seem to be relevant for risk stratification of these patients. However, individual characteristics certainly influence the outcomes of these patients, and they need to be evaluated considering the peculiarities of each population. As highlighted by the ACLARA study [15], patient heterogeneity poses significant challenges in the management of these critically ill subjects. Variations in genetic ancestry and race are linked to disparities in the presentation of ACLF, particularly in its severe forms. It is therefore important to conduct studies addressing clinical features and the course of ACLF in different populations outside Europe and North America.

There is an unmet need in biomarker development to guide therapeutic decisions. Ariza et al. [16] studied urine and plasma neutrophil gelatinase–associated lipocalin (NGAL) concentrations in patients with ACLF. The authors found that urine NGAL was an independent predictor of the development of ACLF and 28‐day transplant‐free mortality. However, up to now, there is no bedside biomarker that can be applied in real‐world clinical practice in those patients [17–19].

Our study primarily aimed to evaluate the frequency of ACLF at different time points in patients with cirrhosis admitted to the ICU and the influence of the clinical course of ACLF on ICU mortality and 28‐day survival. While the limits of therapeutic strategies and potential futility are outside the scope of this study, this is undoubtedly an important point in hepatology critical care. Future studies could be designed to better evaluate this issue.

In agreement with other authors [5, 8], our study reveals that improvement of ACLF significantly enhances short‐term survival. It is therefore important to identify and treat ACLF triggers, particularly infections and alcoholic hepatitis, for ACLF reversal to reduce mortality [20–23].

Given the high mortality rate in critically ill cirrhotic patients, their prognosis in the ICU has been widely studied. Our study found that many ICU–admitted cirrhotic patients, including some patients with multiple OF on admission, were able to be discharged from the ICU. While previous studies suggest a benefit of intensive support for 3 days [24], our findings indicate that extending this support to 7 days may improve even more outcomes and offer a potential path to transplantation for these critically ill patients.

In this study, we chose to exclude patients with severe comorbidities, including HCC beyond Milan’s criteria, extrahepatic malignancy, HIV infection, chronic renal failure requiring dialysis, and heart failure. The rationale for this exclusion was to avoid potential bias that could affect the mortality data.

It is important, however, to highlight that our study has several limitations due to its retrospective design. We were unable to assess longer survival periods as well as the impact of ACLF in the posttransplant follow‐up, as well as the effect of ACLF downstaging on postliver transplantation survival [25]. Given the dismal prognosis of these patients without transplantation, discussions regarding prioritization programs for ACLF can help improve both waiting list mortality and outcomes after transplantation.

In conclusion, ACLF is a serious complication of cirrhosis associated with high mortality during hospitalization. CLIF‐C criteria seem to be more effective for prognostic assessment and early disease management. Prompt infection management combined with 3–7 days of intensive care support may significantly improve clinical outcomes.

**Nomenclature**
AD: Acute decompensation
ACLF: Acute‐on‐chronic liver failure
CLIF‐C: Chronic Liver Failure Consortium
ICU: Intensive care unit
NACSELD: North American Consortium for the Study of End‐Stage Liver Disease
MELD: Model for End‐Stage Liver Disease
OF: Organ failure
LT: Liver transplantation

## Consent

Participant consent was waived because this was a retrospective study, and data were anonymized entirely.

## Conflicts of Interest

The authors declare no conflicts of interest.

## Author Contributions

Paulo Lisboa Bittencourt conceived the study; Liana Codes and Paulo Lisboa Bittencourt supervised data collection; Maria Eduarda Chaves Soares, Bianca Sampaio de Carvalho, Amanda Caroline Silveira e Silva, Myriam Sofia Angeli Guimarães de Oliveira, Fabiola Santos Sousa, Mariana Rebouças de Calasans, Jade de Oliveira Santana, and Lucas Celes Dominguez collected the data; Maria Eduarda Chaves Soares searched for reference articles in the literature for analysis; Liana Codes and Paulo Lisboa Bittencourt drafted the manuscript, and all authors contributed substantially to its revision; Paulo Lisboa Bittencourt takes responsibility for the paper as a whole.

## Funding

No funding was received for this research.

## Data Availability

The data that support the findings of this study are available from the corresponding author upon reasonable request.
